# [^18^F]FDG PET/CT identifies infectious and inflammatory foci in persistent critical illness

**DOI:** 10.1186/s13613-025-01444-0

**Published:** 2025-02-20

**Authors:** Bram van Leer, Jelle L. G. Haitsma Mulier, Cornelis P. van Stee, Kiki M. Demenaga, Riemer H. J. A. Slart, Matijs van Meurs, Andor W. J. M. Glaudemans, Maarten W. N. Nijsten, Lennie P. G. Derde, Janesh Pillay

**Affiliations:** 1 Department of Critical Care, University Medical Centre Groningen, University of Groningen, Groningen, The Netherlands; 2 Department of Nuclear Medicine and Molecular Imaging, University Medical Centre Groningen, University of Groningen, Groningen, The Netherlands; 3 Intensive Care Center, University Medical Center Utrecht, University of Utrecht, Utrecht, The Netherlands; 4 Julius Centre for Health Sciences and Primary Care, Utrecht University, Utrecht, The Netherlands; 5 Biomedical Photonic Imaging Group, Faculty of Science and Technology, University of Twente, Enschede, The Netherlands; 6 Groningen Research Institute for Asthma and COPD (GRIAC), University Medical Center Groningen, University of Groningen, Groningen, The Netherlands; 7 Hanzeplein 1, 9700 RB, TA29, Groningen, 30001 The Netherlands

**Keywords:** Persistent critical illness, [^18^F]FDG PET/CT, Systemic inflammation, Imaging, ICU

## Abstract

**Purpose:**

Some ICU patients remain critically ill despite reversal of the original admission diagnosis, driven by a cascade of events resulting in new and persistent organ failure. Secondary infections and systemic inflammation are important components of this cascade and may be visualised using [^18^F]FDG PET/CT. The aim of this dual centre retrospective study was to assess the ability of [^18^F]FDG PET/CT to identify infectious and inflammatory foci in patients with persistent critical illness and to evaluate its impact on subsequent therapy management.

**Methods:**

We included patients admitted to the ICU between 2017 and 2024, in whom a [^18^F]FDG PET/CT scan was performed ten days or more after ICU admission. [^18^F]FDG PET/CT reports were reviewed for diagnoses, and clinical records were reviewed to determine if this diagnosis was new, which diagnostics were performed before the PET/CT, and which therapeutic changes were made directly after the PET/CT. The relation between inflammatory parameters and [^18^F]FDG PET/CT findings were studied using t-test or ANOVA.

**Results:**

Forty-seven patients with persistent critical illness were included from two university medical centres. The median interval between admission and PET/CT was 21 days (IQR 14–28). In 43 patients (91%) a potential infectious or inflammatory focus was detected, of which 34 (72%) were previously unknown. The [^18^F]FDG PET/CT was utilized late in the diagnostic work-up since a median of 7 (IQR 6.0–8.0) diagnostic procedures were performed prior to the PET/CT. In 26 (55%) patients therapy change was reported within 48 h after the PET/CT.

**Conclusion:**

[^18^F]FDG PET/CT detected a considerable number of (new) infectious and inflammatory foci in patients with persistent critical illness, often followed by a change in therapy. Further research is needed to establish the role of [^18^F]FDG PET/CT in these patients.

## Background

In intensive care medicine, reversal of the original admission diagnosis can be insufficient to improve outcomes, and patients may remain dependent on organ support therapies for a prolonged period of time. In the literature this is known as ‘persistent critical illness’ which is defined as an ICU stay of 10 days or more [1–3]. In persistent critical illness, the outcome is no longer determined by the admission diagnosis, but by a cascade of events resulting in new and persistent organ failure.

Though relatively few patients (± 5%) have an ICU stay of ≥ 10 days, these patients consume significant medical resources, occupying 30–55% of ICU beds [1, 4]. Outcomes for these patients are poor, with hospital mortality estimated to be around 25% [1]. Moreover, survivors are more likely to be discharged to rehabilitation or long-term care facilities.

ICU-acquired infections and inflammation play an important role in the development of persistent critical illness [5, 6]. Altered immune responses, along with the prolonged use of infection-prone interventions such as central venous catheters and invasive ventilation, contribute to susceptibility to nosocomial secondary infections and persistent inflammation [6–10]. This is reflected by the observation that more than 50% of patients with persistent critical illness develop a new septic episode [5]. Dampened systemic inflammatory responses to local infections mask overt clinical signs of infection and could delay the diagnosis [11]. In these patients, timely identification of infectious and inflammatory foci is essential to reduce resource use and improve outcomes.

The presence and location of (unknown) infections and inflammatory foci can be identified with [^18^F]-fluorodeoxyglucose positron emission tomography ([^18^F]FDG PET/CT). During an inflammatory process the glucose consumption is raised due to the influx of lymphocytes (mainly neutrophils and macrophages/monocytes) and upregulation of glucose transporters such as GLUT1 and GLUT3 caused by the cytokine release. Furthermore, the cytokines increases the glucose metabolism and strengthen the affinity of glucose for the glucose transporters. [^18^F]FDG mimics the function of glucose and is metabolised likewise. However, [^18^F]FDG cannot fully be metabolised and is therefore trapped in the cell. This allows imaging of the accumulation of [^18^F]FDG, which is increased in inflamed tissue. This whole body imaging modality is routinely used outside of the critical care setting in patients with bacteraemia, fever of unknown origin, endocarditis, vasculitis, vascular graft infections, peripheral bone infections and prosthetic joint infections [12, 13]. However, [^18^F]FDG PET/CT is infrequently used in ICU patients and has only been described in a few single centre observational studies with small sample sizes [14–19]. 

In this study, we aimed to investigate the ability of [^18^F]FDG PET/CT to identify (unknown) infectious and inflammatory foci in patients with persistent critical illness. In addition, we examined the number and type of diagnostic procedures preceding the PET/CT scan and whether the findings on the PET/CT resulted in change of therapy.

## Methods

A retrospective study was performed in two university medical centres in the Netherlands (University Medical Center Utrecht and University Medical Center Groningen). All adults in whom an [^18^F]FDG PET/CT scan was performed after at least 10 days of ICU admission, but before ICU discharge, between December 2017 and May 2024 were included. Only the first eligible [^18^F]FDG PET/CT scan for each patient was included. Data were extracted by three trained investigators from clinical records and PET/CT reports. [^18^F]FDG PET/CT reports provided by nuclear physicians as part of clinical routine were used for the analysis. A focus or localization was considered new if it was not recorded in the patient’s electronic health records of the 7 days preceding the [^18^F]FDG PET/CT scan.

The study was approved by the ethical board of the University Medical Center Groningen and the need for informed consent was waived (IRB number: 2023/506).

For analysis of diagnostic procedures conducted during ICU admission prior to the [^18^F]FDG PET/CT scan, diagnostic procedures were recorded, regardless of matching indications. For imaging procedures the details of the last scan were extracted. Cultures from blood, sputum, wounds, catheter tips, cerebrospinal fluid or feces were reported, if obtained within the 7 days preceding the [^18^F]FDG PET/CT. Blood cultures were considered contaminated if identified as such in the clinical reports or if only a single culture showed growth of an organism typically associated with contamination (e.g. *CNS*, S. *hominis or S. epidermidis*). Contaminated cultures were excluded from the study. Each type of diagnostic procedure was recorded as ‘yes’ or ‘no’ only once, even if performed multiple times, to focus on the spectrum of diagnostic procedures rather than the total number of procedures.

Change in therapy was defined as any new therapeutic decision reported to be made within 48 h after the [^18^F]FDG PET/CT. This included, but was not limited to, initiating, continuing or discontinuing antibiotic or steroid therapy, performing (surgical) drainage of fluid collections, and restricting or withdrawal of interventions, like mechanical ventilation or extracorporeal membrane oxygenation.

Findings are reported as descriptives. For continuous data median and inter quartile range (IQR) was used. Categorical data is reported as frequency and percentages. The relations between inflammatory markers (CRP, leukocytes and temperature) and [^18^F]FDG PET/CT findings were studied using an independent T-test or ANOVA. Data was analysed using SPSS 29, IBM corp. Armonk, USA.

## Results

### Patients

Forty-seven patients with a median ICU stay of 39 days (IQR 25–61) were included. The primary reason for admission was respiratory failure (18, 38%), post-surgery (9, 19%), septic shock (6, 13% ), cardiogenic shock (3, 6%) and status epilepticus (3, 6%). Respiratory support was the most common primary reason for prolonged length of ICU stay (*n* = 26, 55%). During the [^18^F]FDG PET/CT procedure most patients received mechanical ventilation (*n* = 39, 83%) and/or circulatory support (*n* = 35, 75%) (Table 1).

**Table 1:** Baseline patient characteristics

	*n* (%) or median [IQR]
Total number of patients	47
Age	59 [31–69]
Male	30 (64)
Mechanically ventilated	39 (83)
Tracheostomy	13 (28)
Circulatory support	35 (75)
Renal replacement therapy	16 (34)
Acute kidney failure	27 (57)
SOFA-score at time of PET request	7 [5–9]
ICU mortality	10 (21)
ICU length of stay (days)	39 [25–61]
ICU day of PET/CT	21 [14–28]
Measurements were taken at the time of [^18^F]FDG PET/CT unless otherwise stated.	

### Diagnostic procedures preceding [^18^F]FDG PET/CT

A median of 7 types of diagnostic procedures (IQR 6.0–8.0, minimum 3, maximum 10) were performed per patient during ICU admission prior to the [^18^F]FDG PET/CT scan. All patients received conventional chest X-ray prior to [^18^F]FDG PET/CT scan and in most patients (*n* = 41, 87%) at least one CT scan was performed. The median time from the last CT scan to [^18^F]FDG PET/CT was 14 days (IQR 5–20). A chest CT-scan was performed in 10 patients (24%), abdominal CT-scan in 8 patients (20%) and 9 patients received both (22%). Cranial CT was performed in 6 patients (15%). Additionally, total body CT scan (i.e. cranial, chest, and abdomen) was performed in 4 patients (10%). Other types of CT scans performed included those to image vessels, spine, extremities or for CT guided thoracic drainage. In more than half of the patients (*n* = 26, 55%) radiological ultrasound was performed, mostly for suspicion of fluid collections or thrombosis. In 4 (9%) patients, an earlier [^18^F]FDG PET/CT scan was performed within 9 days of ICU admission. In 3 of these patients a new focus was reported in the subsequent [^18^F]FDG PET/CT as included in this study, with localizations in the lungs in 2 patients and around an orthopaedic screw in 1. Blood cultures were obtained before the PET/CT in most patients (*n* = 45, 96%), of which 25 (55%) were positive. *Staphylococcus epidermidis*, *Staphylococcus hominis*, *Enterococcus faecalis*, *Enterococcus faecium* and *Staphylococcus aureus* were most frequently reported. Sputum cultures were obtained in 46 patients of which 18 (39%) were positive. Bronchoalveolar lavage (BAL) was performed in 13 patients (28%), resulting in positive BAL fluid cultures in 5 patients (38%). All diagnostic procedures performed prior to [^18^F]FDG PET/CT are listed in Table 2.

**Table 2:** Diagnostic procedures performed prior to the investigated [^18^F]FDG-PET/CT scan

Imaging	*n* (%) or median [IQR]
PET/CT < 10 days of ICU admission	4 (9)
Time between last and included PET/CT	16 [9–18] days
Last CT prior to PET/CT	41 (87)
Chest	10 (24)
Chest and Abdomen	9 (22)
Abdomen	8 (20)
Cerebrum	6 (15)
Head, Chest and Abdomen	4 (10)
Other	4 (10)
Time between last CT scan and PET/CT	14 [5–20] days
Ultrasound	26 (55)
MRI	5 (11)
X-ray	
Chest	47 (100)
Abdomen	8 (17)
Limbs	2 (4)
Endoscopy	5 (11)
**Cultures**	
Blood	45 (96)
Positive	25 (55)
Sputum	46 (98)
Positive	18 (39)
Tip CVC	28 (60)
Positive	17 (61)
Fecal	6 (13)
Positive	0 (0)
Wound	20 (43)
Positive	10 (50)
Lumbar	2 (4)
Positive	0 (0)
BAL	13 (28)
Positive	5 (38)
Diagnostic surgery	3 (6)
Positive	1 (33)
**Other**	
Biopsy	6 (13)
Urine test	13 (28)
Positive	3 (23)

Diagnostic procedures were scored regardless of matching indications. For imaging procedures only the last scan was evaluated. Cultures performed within 7 days preceding the PET/CT were obtained. Each type of diagnostic procedure was recorded only once, even if performed multiple times. CVC = central venous catheter; BAL = Bronchoalveolar lavage

### [^18^F]FDG PET/CT > days 10 after ICU admission

[^18^F]FDG PET/CT was performed at a median of 21 days of ICU admission (IQR 14–28) (Table 1). Persistent systemic inflammation without sufficient clarification was the main reason for performing a [^18^F]FDG PET/CT (*n* = 39, 83%) (Table 3). In 6 (13%) patients a PET/CT was performed to confirm a suspected focal infection (*n* = 3; abdominal, infected left ventricular assist device, and endocarditis/mediastinitis) or to study dissemination of an already known infection (*n* = 3). In 43 patients (91%) an inflammatory or infectious focus was identified. In the majority of patients (*n* = 34, 72%) the foci were identified as new. These new foci were found mainly in the chest (*n* = 15, 44%) and abdomen (*n* = 6, 18%). Other localizations were joint, bone, muscle, around orthopaedic screws, infected hematomas, infected thrombi, pituitary gland and nasal sinuses (Table 3). The [^18^F]FDG PET/CT findings were followed by change in therapy in 26 patients (55%). Changes were made mainly in antibiotic therapy. In 8 (17%) patients antibiotic therapy was stopped, in 6 (13%) patients the type of antibiotic was changed, in 1 (2%) patient the duration of antibiotic treatment was prolonged, and in 1 (2%) patient antibiotic therapy was initiated based on the [^18^F]FDG PET/CT. In 5 (11%) patients a bronchoalveolar lavage was performed after the PET/CT. Other interventions that were performed after the PET/CT were surgical interventions in 4 (8%), drainage of fluid collections in 4 (8%), and start of steroids in 3 (6%) patients. Only in 2 (4%) patients follow-up imaging was performed. See Table 3 for all changes in therapy management. In the patients with a negative PET/CT result therapy changes were reported in 2 (50%) patients. One patient was weaned from the ventilator and antibiotics were stopped and in the other patient the antibiotic drug was changed.

**Table 3:** [^18^ F]FDG PET/CT indication, diagnosis, localization and therapy change

		*n* (%)
**PET/CT indication**		
Persistent systemic inflammation		39 (83)
Clinical suspicion of an infection		6 (13)
Clinical suspicion of an malignancy		2 (4)
**PET/CT diagnoses**		
No focus		4 (8)
Any focus		43 (91)
New infectious focus		27 (63)
New inflammatory focus		6 (14)
New infectious and inflammatory focus		1 (2)
**Localization of new PET/CT findings**		
Chest		15 (44)
Abdominal		6 (18)
Multiple		3 (9)
Other		10 (29)
**Change in therapy management**		
Any change		26 (55)
Antibiotics		16 (34)
Steroids		3 (6)
Surgical intervention		4 (9)
Drainage of fluid collection		4 (9)
Punction		1 (2)
Removal central venous catheter		1 (2)
Weaning of mechanical ventilation		1 (2)
Bronchoalveolar lavage		5 (11)
Follow-up imaging		2 (4)
Withdrawal / Limitation of care		2 (4)

Infectious or inflammatory foci were considered new if it was not recorded in the patient’s electronic health records of the 7 days preceding the [^18^F]FDG PET/CT scan. Change in therapy was defined as any new therapeutic decision reported to be made within 48 h after the [^18^F]FDG PET/CT

**Table 4:** Inflammatory status at time of [^18^F]FDG PET/CT

		All	New findings	No new findings	Sig.
CRP mg/L	*Median [IQR]*	141 [82–200]	131 [71–205]	149.0 [121–218]	0.37
CRP > 100	*n (%)*	33 (70)	21 (62)	12 (92)	
Leukocytes 10^9^/L	*Median [IQR]*	11.6 [8.0-16.2]	10.4 [7.8–16.0]	14.9 [10.8–16.8]	0.85
< 4	*n (%)*	4 (8)	2 (6)	2 (15)	
> 11	*n (%)*	26 (55)	16 (47)	10 (77)	
Body temperature °C	*Median [IQR]*	37.2 [36.6–37.9]	37.3 [36.7–38.1]	37.1 [36.6–37.6]	0.50
> 38.0	*n (%)*	9 (19)	8 (24)	1 (8)	
Antibiotic therapy	*n (%)*	40 (85)	28 (82)	12 (92)	
Steroid therapy	*n (%)*	9 (19)	6 (18)	3 (23)	

The relations between inflammatory markers and [^18^F]FDG PET/CT findings were studied using an independent T-test or ANOVA

### Inflammatory parameters

One or more abnormal inflammatory parameters (i.e. elevated CRP, leukocytes or temperature) were present in all but five patients (*n* = 42, 89%) (Table 4). Median CRP levels were 131 mg/L (IQR 71–205) and 149 mg/L (121–218, *p* = 0.37) for patients with and without new findings, respectively. For leukocytes this was 10.4*10^9^/L (7.8–16.0) and 14.9*10^9^/L (10.8–16.8, *p* = 0.85) and for body temperature 37.3 °C (36.7–38.1) and 37.1 °C (36.6–37.6, *p* = 0.50). On the day of the PET/CT 28 patients (82%) with new findings and 12 patients (92%) without new findings received antibiotic therapy. For steroid therapy this was 6 (18%) and 3 (23%).

## Discussion

This dual centre retrospective study, the largest of its kind, investigated the application of [^18^F]FDG PET/CT to identify infectious or inflammatory foci in patients with persistent critical illness treated on the ICU. Remarkably, [^18^F]FDG-PET/CT identified an infectious or inflammatory focus in 92% of patients, with 72% of those reported to be new. In more than half of the included patients, PET/CT contributed to a change in therapy, highlighting its potential role in guiding clinical decisions within the ICU.

In most instances, [^18^F]FDG PET/CT was utilized late in the diagnostic workup, as evidenced by the fact that [^18^F]FDG PET/CT was preceded by a large number of alternate diagnostic procedures. Interestingly, although extensive thoracic diagnostic procedures were performed in most patients (chest X-ray, CT and BAL), PET/CT frequently revealed new thoracic localizations. This suggests an important added value of [^18^F]FDG PET/CT.

The yield of new findings (72%) reported in this study is higher than reported previously. Retrospective studies focusing on patients with septic shock of unknown origin and bloodstream infections of unknown origin, showed new findings in 27% and 37%, respectively [14, 16]. In the only prospective study performed, investigating [^18^F]FDG PET/CT with CT angiography in suspected severe sepsis patients with no definite diagnosis, PET/CT added crucial information in 29% of the patients [17]. Differences in yield between this study and previous studies could be explained by the timing of the PET/CT, resulting in a larger number of patients included in the current study with dampened systemic inflammation and increased likelihood of nosocomial infections. Secondly, different definitions of new findings were used. Kluge et al. reported [^18^F]FDG PET/CT scans that were essential for diagnosis, but did not specify what was meant by essential [14]. Pijl et al. reported the number of patients with an new infectious focus but excluded suspected, but not established, foci [16]. In the current study, all findings not documented as diagnosis in the electronic health record prior to the PET/CT were classified as new, which, in our opinion, better reflects clinical practice. The contribution to therapy change in our study was comparable with previous studies [14–17]. 

Outside the ICU, fever or inflammation of unknown origin (FUO/IUO) is a common problem and although its aetiology and definition differ from infections seen in persistent critically-ill patients, the nature of the diagnostical problem shows similarities. FUO/IUO is a well-established indication for [^18^F]FDG PET/CT and large studies show an added value of PET/CT in 60-70% of these cases [13]. Furthermore, for FUO/IUO, PET/CT showed better performance than contrast enhanced CT scans [20]. This further underscores the potential of [^18^F]FDG PET/CT in patients with persistent critical illness.

**Fig. 1:**
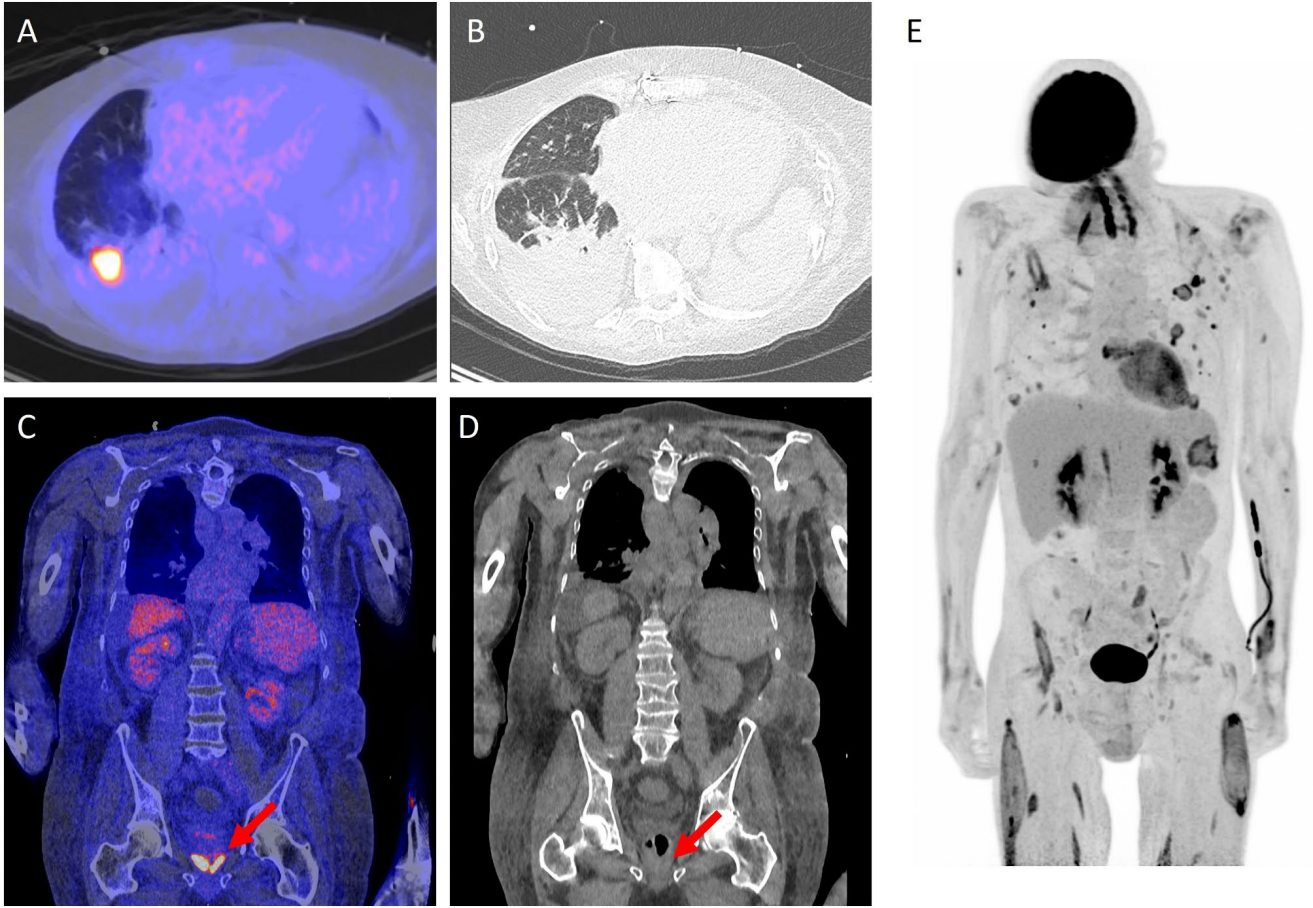
There are several conceivable advantages of [^18^F]FDG PET/CT over conventional imaging. Image **A** and **B** showing an example of the distinctive capability of the [^18^F]FDG PET/CT. **A** shows a fusion image of [^18^F]FDG PET/CT and low-dose CT and **B** an high resolution chest CT of a patient with an Aspergillus infection. The suspected aspergilloma is concealed on the high resolution CT due to the lung fluids and consolidation. Image **C** and **D** (fusion image of [^18^F]FDG PET/CT and low-dose CT and low-dose CT only, respectively) are examples of the ability to image inflammation while no concurrent abnormalities are visualized, in this case prostatitis (red arrows). Image **E**, a maximized intensity projection of [^18^F]FDG PET, shows a patient with lung infections and multiple muscle abscesses after MRSA sepsis. This is an illustrative example of a relevant dissemination investigation

There are several conceivable advantages of [^18^F]FDG PET/CT in critically-ill patients. Conventional imaging modalities depict anatomical abnormalities caused by pathological processes. However, [^18^F]FDG PET/CT displays the patho(physio)logical processes themselves due to their increased glycolytic rate, and even before anatomical changes occur. Therefore, it is plausible that this technique is more sensitive to image infectious or inflammatory foci as can be seen in Fig. 1. PET/CT is able to discriminate within abnormalities (Fig. 1A and B) without the necessity for anatomical changes (Fig. 1C and D). Furthermore, when used for dissemination purposes it has the added advantage of whole body imaging (Fig. 1E). Since unknown infection or inflammation is the main indication to perform a [^18^F]FDG PET/CT scan in ICU patients, whole body imaging is essential [18, 19]. 

Our study has limitations. First, it concerns a retrospective study exploring the utility of [^18^F]FDG PET/CT, which is inherently limited by its design and therefore should be interpreted with caution. However, to our knowledge, it is the first multi-centre and largest retrospective study reporting the potential value of [^18^F]FDG PET/CT imaging in critically ill patients [17, 18]. Secondly, this study has a risk of selection bias. PET/CT was performed at the discretion of the treating clinicians, therefore a high suspicion of an infection or inflammation was likely present. However, the identification of a substantial number of new findings shows the potential of [^18^F]FDG PET/CT in this patient population. This study shows that in the context of persistent critical illness and clinical suspicion of infection/inflammation, [^18^F]FDG PET/CT could be useful. Third, the study does not allow for a direct comparison of the performance of [^18^F]FDG PET/CT with other diagnostic tools as they were not performed on the same date. Due to the large interval between initial CT and PET/CT (median 14 days), it is likely that PET/CT identified processes that were not yet present on previously performed CT scans. However, the studies from Mandry et al. and Buchrits et al. both suggest better performance of [^18^F]FDG PET/CT when directly compared to contrast enhanced CT [17, 20]. Finally, the reported changes in therapy cannot directly be attributed to the results of the PET/CT scan. While it is conceivable that the results influenced decision-making directly after the PET/CT scan, it cannot be stated with certainty. We therefore reported all clinical decisions made within 48 h after the PET/CT scan.

PET/CT scans are considered complex procedures for critically ill patients [21]. However, with new Large-Field-of-View PET/CT camera systems, scans can be acquired within a couple of minutes [22]. This corresponds to scan times of contrast enhanced CT scans [23, 24]. Well-structured preparation protocols and better awareness among ICU personnel could increase adoption of [^18^F]FDG PET/CT imaging in selected critically ill patients [19, 25]. 

Future studies should focus on clinical indications, optimal patient preparation, optimal ICU [^18^F]FDG PET/CT protocols and timing of [^18^F]FDG PET/CT in critically ill patients. Furthermore, routinely used inflammatory parameters appear not to be helpful for patient selection, from our study. Future studies should investigate if biomarkers can predict those with high diagnostic yield from PET/CT. In addition, direct comparisons between [^18^F]FDG PET/CT and contrast-enhanced CT should be investigated.

In conclusion, [^18^F]FDG PET/CT detected a considerable number of new infectious and inflammatory foci in ICU patients with persistent critical illness, often followed by a change in therapy. Further research is needed to establish the role of [^18^F]FDG PET/CT in these patients.

## Data Availability

The datasets used and/or analysed during the current study are available from the corresponding author on reasonable request.

## Abbreviations

ICU: Intensive Care Unit
[^18^F]FDG PET/CT: [^18^F]–fluorodeoxyglucose positron emission tomography/computed tomography
IQR: Inter Quartile Range
FUO: Fever of Unknown Origin
IUO: Inflammation of Unknown Origin

## References

[1] Iwashyna TJ, Hodgson CL, Pilcher D, Bailey M, van Lint A, Chavan S et al. Timing of onset and burden of persistent critical illness in Australia and New Zealand: a retrospective, population-based, observational study. Lancet Respir Med [Internet]. 2016 Jul 1 [cited 2023 Apr 13];4(7):566–73. Available from: https://pubmed.ncbi.nlm.nih.gov/27155770/10.1016/S2213-2600(16)30098-4 27155770

[2] Iwashyna TJ, Hodgson CL, Pilcher D, Bailey M, Bellomo R. Persistent critical illness characterised by Australian and New Zealand ICU clinicians. Crit Care Resusc. 2015;17(3):153–e15316. 26282252

[3] Ohbe H, Satoh K, Totoki T, Tanikawa A, Shirasaki K, Kuribayashi Y et al. Definitions, epidemiology, and outcomes of persistent/chronic critical illness: a scoping review for translation to clinical practice. Critical Care 2024 28:1 [Internet]. 2024 Dec 28 [cited 2025 Jan 9];28(1):1–15. Available from: https://ccforum.biomedcentral.com/articles/10.1186/s13054-024-05215-4 10.1186/s13054-024-05215-4 PMC11681689 39731183

[4] Bagshaw SM, Stelfox HT, Iwashyna TJ, Bellomo R, Zuege D, Wang X. Timing of onset of persistent critical illness: a multi-centre retrospective cohort study. Intensive Care Med [Internet]. 2018 Dec 1 [cited 2023 Apr 13];44(12):2134–44. Available from: https://pubmed.ncbi.nlm.nih.gov/30421256/10.1007/s00134-018-5440-1 30421256

[5] Darvall JN, Boonstra T, Norman J, Murphy D, Bailey M, Iwashyna TJ et al. Persistent critical illness: baseline characteristics, intensive care course, and cause of death. Vol. 21, Critical Care and Resuscitation •. 2019. 31142241

[6] Nedeva C, Menassa J, Puthalakath H. Sepsis: inflammation is a necessary evil. Front Cell Dev Biol. 2019;7(JUN):447614. 10.3389/fcell.2019.00108 PMC6596337 31281814

[7] Kalb TH, Lorin S. Infection in the chronically critically ill: unique risk profile in a newly defined population. Crit Care Clin. 2002;18(3):529–52. 12140912 10.1016/s0749-0704(02)00009-x

[8] Lafuente Cabrero E, Terradas Robledo R, Civit Cuñado A, García Sardelli D, Hidalgo López C, Giro Formatger D et al. Risk factors of catheter- associated bloodstream infection: Systematic review and meta-analysis. PLoS One [Internet]. 2023 [cited 2024 May 24];18(3):e0282290. Available from: https://journals.plos.org/plosone/article?id=10.1371/journal.pone.0282290 10.1371/journal.pone.0282290 PMC10035840 36952393

[9] Pitiriga V, Kanellopoulos P, Bakalis I, Kampos E, Sagris I, Saroglou G et al. Central venous catheter-related bloodstream infection and colonization: the impact of insertion site and distribution of multidrug-resistant pathogens. Antimicrob Resist Infect Control [Internet]. 2020 Dec 1 [cited 2024 May 24];9(1):1–8. Available from: https://aricjournal.biomedcentral.com/articles/10.1186/s13756-020-00851-1 10.1186/s13756-020-00851-1 PMC7708904 33261661

[10] Alp E, Voss A. Ventilator associated pneumonia and infection control. Ann Clin Microbiol Antimicrob [Internet]. 2006 Apr 6 [cited 2024 May 24];5(1):1–11. Available from: https://ann-clinmicrob.biomedcentral.com/articles/10.1186/1476-0711-5-7 10.1186/1476-0711-5-7 PMC1540438 16600048

[11] Pfortmueller CA, Meisel C, Fux M, Schefold JC. Assessment of immune organ dysfunction in critical illness: utility of innate immune response markers. Intensive Care Medicine Experimental [Internet]. 2017 Dec 1 [cited 2024 May 31];5(1):1–16. Available from: https://icm-experimental.springeropen.com/articles/10.1186/s40635-017-0163-0 10.1186/s40635-017-0163-0 PMC5653680 29063386

[12] Glaudemans AWJM, Gheysens O. Expert opinions in nuclear medicine: finding the holy grail in infection imaging. Front Med (Lausanne). 2023;10:320. 10.3389/fmed.2023.1149925 PMC10008957 36923013

[13] van Rijsewijk ND, IJpma FFA, Wouthuyzen-Bakker M, Glaudemans AWJM. Molecular Imaging of Fever of unknown origin: an update. Semin Nucl Med. 2023;53(1):4–17. 35902280 10.1053/j.semnuclmed.2022.07.002

[14] Kluge S, Braune S, Nierhaus A, Wichmann D, Derlin T, Mester J et al. Diagnostic value of positron emission tomography combined with computed tomography for evaluating patients with septic shock of unknown origin. J Crit Care [Internet]. 2012 [cited 2023 Apr 18];27(3):316.e1-316.e7. Available from: https://pubmed.ncbi.nlm.nih.gov/22176803/10.1016/j.jcrc.2011.10.004 22176803

[15] Simons KS, Pickkers P, Bleeker-Rovers CP, Oyen WJG, Van Der Hoeven JG. F-18-fluorodeoxyglucose positron emission tomography combined with CT in critically ill patients with suspected infection. Intensive Care Med [Internet]. 2010 Mar [cited 2023 Jan 6];36(3):504–11. Available from: https://pubmed.ncbi.nlm.nih.gov/19847397/10.1007/s00134-009-1697-8 PMC2820225 19847397

[16] Pijl JP, Londema M, Kwee TC, Nijsten MWN, Slart RHJA, Dierckx RAJO et al. FDG-PET/CT in intensive care patients with bloodstream infection. Crit Care [Internet]. 2021 Dec 1 [cited 2022 Jul 11];25(1). Available from: https://pubmed.ncbi.nlm.nih.gov/33827655/10.1186/s13054-021-03557-x PMC8028784 33827655

[17] Mandry D, Tatopoulos A, Chevalier-Mathias E, Lemarié J, Bollaert PE, Roch V et al. ^18^F-fluorodeoxyglucose positron emission tomography combined with whole-body computed tomographic angiography in critically ill patients with suspected severe sepsis with no definite diagnosis. Eur J Nucl Med Mol Imaging [Internet]. 2014 Oct 1 [cited 2023 Apr 18];41(10):1924–30. Available from: https://pubmed.ncbi.nlm.nih.gov/24848788/10.1007/s00259-014-2804-9 24848788

[18] van Hulst A, van Rijk M, Bavelaar-Croon C, Tjan D. The value of F-18-fluorodeoxyglucose positron emission tomography (FDG-PET/CT) in the intensive care unit: a review. Neth J Crit Care. 2019;27(03):108–14.

[19] van Leer B, van Rijsewijk ND, Nijsten MWN, Slart RHJA, Pillay J, Glaudemans AWJM. Practice of 18F-FDG-PET/CT in ICU patients: a systematic review. Semin Nucl Med. 2023. 10.1053/j.semnuclmed.2023.05.003 37258380

[20] Buchrits S, Gafter-Gvili A, Eynath Y, Bernstine H, Guz D, Avni T. The yield of F18 FDG PET-CT for the investigation of fever of unknown origin, compared with diagnostic CT. Eur J Intern Med [Internet]. 2021 Nov 1 [cited 2023 Oct 6];93:50–6. Available from: http://www.ejinme.com/article/S0953620521002648/fulltext 10.1016/j.ejim.2021.07.014 34420847

[21] Hess S. [18F]FDG-PET/CT in patients with bacteremia: clinical impact on patient management and outcome. Front Med (Lausanne). 2023;10:1157692. 37064040 10.3389/fmed.2023.1157692 PMC10095558

[22] Slart RHJA, Tsoumpas C, Glaudemans AWJM, Noordzij W, Willemsen ATM, Borra RJH et al. Long axial field of view PET scanners: a road map to implementation and new possibilities. Eur J Nucl Med Mol Imaging [Internet]. 2021 Dec 1 [cited 2022 Aug 26];48(13):4236–45. Available from: https://pubmed.ncbi.nlm.nih.gov/34136956/10.1007/s00259-021-05461-6 PMC8566640 34136956

[23] Alberts I, Sari H, Mingels C, Afshar-Oromieh A, Pyka T, Shi K et al. Long-axial field-of-view PET/CT: perspectives and review of a revolutionary development in nuclear medicine based on clinical experience in over 7000 patients. Cancer Imaging 2023 23:1 [Internet]. 2023 Mar 18 [cited 2024 May 31];23(1):1–15. Available from: https://cancerimagingjournal.biomedcentral.com/articles/10.1186/s40644-023-00540-3 10.1186/s40644-023-00540-3 PMC10024603 36934273

[24] Roya M, Mostafapour S, Mohr P, Providência L, Li Z, van Snick JH et al. Current and Future Use of Long Axial Field-of-View Positron Emission Tomography/Computed Tomography Scanners in Clinical Oncology. Cancers (Basel) [Internet]. 2023 Nov 1 [cited 2024 May 31];15(21). Available from: /pmc/articles/PMC10648837/10.3390/cancers15215173 PMC10648837 37958347

[25] van Snick JH, van Leer B, Nijsten MWN, Pillay J, Slart RHJA, Glaudemans AWJM, et al. Long axial field of view PET/CT in critically ill patients: lessons from a case report. Front Med (Lausanne). 2023;10:1347791. 38239612 10.3389/fmed.2023.1347791 PMC10794769

